# Treatment burden for people experiencing homelessness with a recent non-fatal overdose: a questionnaire study

**DOI:** 10.3399/BJGP.2022.0587

**Published:** 2023-07-11

**Authors:** Caitlin Jones, Frances S Mair, Andrea E Williamson, Andrew McPherson, David T Eton, Richard Lowrie

**Affiliations:** GP registrar and Scottish Clinical Research Excellence Development Scheme (SCREDS) post holder 2021–2023;; School of Health and Wellbeing, University of Glasgow, Glasgow, UK.; School of Health and Wellbeing, University of Glasgow, Glasgow, UK.; Glasgow Pharmacy Services, NHS Greater Glasgow and Clyde, Glasgow, UK.; Social and Behavioural Science, National Cancer Institute, National Institutes of Health, Bethesda, MD, US.; Glasgow Pharmacy Services, NHS Greater Glasgow and Clyde, Glasgow, UK.

**Keywords:** burden of illness, drug overdose, homelessness, multimorbidity, primary health care, surveys and questionnaires

## Abstract

**Background:**

People experiencing homelessness (PEH) who have problem drug use have complex medical and social needs, with barriers to accessing services and treatments. Their treatment burden (workload of self-management and impact on wellbeing) remains unexplored.

**Aim:**

To investigate treatment burden in PEH with a recent non-fatal overdose using a validated questionnaire, the Patient Experience with Treatment and Self-management (PETS).

**Design and setting:**

The PETS questionnaire was collected as part of a pilot randomised control trial (RCT) undertaken in Glasgow, Scotland; the main outcome is whether this pilot RCT should progress to a definitive RCT.

**Method:**

An adapted 52-item, 12-domain PETS questionnaire was used to measure treatment burden. Greater treatment burden was indicated by higher PETS scores.

**Results:**

Of 128 participants, 123 completed PETS; mean age was 42.1 (standard deviation [SD] 8.4) years, 71.5% were male, and 99.2% were of White ethnicity. Most (91.2%) had >5 chronic conditions, with an average of 8.5 conditions. Mean PETS scores were highest in domains focusing on the impact of self-management on wellbeing: physical and mental exhaustion (mean 79.5, SD 3.3) and role and social activity limitations (mean 64.0, SD 3.5) Scores were higher than those observed in studies of patients who are not homeless.

**Conclusion:**

In a socially marginalised patient group at high risk of drug overdose, the PETS showed a very high level of treatment burden and highlights the profound impact of self-management work on wellbeing and daily activities. Treatment burden is an important person-centred outcome to help compare the effectiveness of interventions in PEH and merits inclusion in future trials as an outcome measure.

## INTRODUCTION

The number of people experiencing homelessness (PEH) in Scotland was 42 149 in 2020/2021,^1^ and this population have a high rate of premature mortality; in Scotland, over half of deaths among PEH are drug related.^2^^–^^4^ Scotland has the highest whole-population rate of drug deaths in Europe,^5^ and in 2020 the rate of drug-related deaths was 3.7 times higher than the rest of the UK.^5^^,^^6^ The *Hard Edges Scotland* report described the complex overlapping issues that people may experience, described as severe and multiple disadvantage. The experience of homelessness is one of those, and it describes how PEH and especially those who used ‘hard drugs’ were likely to have the most complex needs, with high rates of poor mental health, involvement in ‘street culture’, and services that did not meet their needs.^7^

Treatment burden describes the workload of treatment and self-management and its impact on patient functioning and wellbeing.^8^^–^^10^ Workload includes activities a patient has do to care for their health, for example, taking medications, keeping medical appointments, monitoring health, and enacting lifestyle changes.^11^^–^^13^ The impact is the patient’s perception of the effect of the workload on role, social, and psychological functioning.^11^^–^^13^ Previous studies have found that PEH are more likely to live with multimorbidity compared with the general population;^14^^–^^16^ however, little is known about their treatment burden.

The Pharmacist Homeless Outreach Engagement Non-medical Independent prescribing Rx (PHOENIx)^17^^,^^18^ pilot randomised control trial (RCT) involved offering a weekly outreach health and social care intervention to PEH who have had a recent non-fatal drug overdose in the preceding 6 months, compared with care as usual. The intervention included weekly visits by the PHOENIx team (a prescribing pharmacist and a charity support worker) for 9 months. Exploratory outcomes include health-related quality of life, changes to treatment burden, and whether the intervention can reduce overdoses. As part of baseline data collection, a slightly modified version of the Patient Experience with Treatment and Self-management (PETS) was used to assess perceived treatment burden.^19^ Although PETS has been used to assess treatment burden in socially marginalised patient groups,^20^ this is the first time it has been used with PEH.

The aim of this study was to understand the treatment burden of PEH in an urban centre in Glasgow, to address a key evidence gap, inform service provision for health care, and ascertain what can be learned for further research.

**Table:** How this fits in

This study aimed to address the knowledge gap of treatment burden among people experiencing homelessness (PEH), who are known to experience high levels of multimorbidity. PEH reported high levels of treatment burden, especially in domains focusing on the impact of self-management on activities and wellbeing. This adds to barriers for PEH maintaining their health and participating effectively in their self-care. Understanding more about treatment burden in PEH can help inform future tailored healthcare interventions and enable evaluation of the impact of such interventions.

## METHOD

### Participant identification and recruitment

Participants were recruited as part of the PHOENIx trial (trial registration: ISRCTN10585019). All participants were homeless adults aged ≥18 years who had a non-fatal drug overdose in the preceding 6 months. Patients were recruited by independent researchers from a range of venues in Glasgow, Scotland, that included the streets, temporary accommodation, and homeless drop-in centres. Participants were randomised 1:1 to the intervention or control group. Data collected included: demographics; living situation; physical and mental health conditions; illicit drug use; diet; social activities; prescribed medications; physiological measures; blood-borne virus status; treatment burden (PETS); psychological distress (Patient Health Questionnaire 4); and quality of life (EQ-5D-5L). Of 128 participants recruited into the trial, 123 (96.1%) responded to the PETS. All data were collected in person at a face-to-face interview conducted by a research nurse.

Participants in the intervention group were offered weekly visits by the PHOENIx team for 6–9.5 months; these visits used a holistic approach to help patients with a wide range of issues, including physical and mental health problems, problem substance misuse, housing, and welfare. All patients (intervention and control) continued to receive care as usual. Usual care comprised all NHS primary and acute care services, community services (for example, community pharmacies), and social care services. All patients were followed up at 6 and then 9 months to repeat the health assessment, including the PETS. The analyses reported here are of the baseline assessment only (pre-intervention). The results of the PHOENIx trial will be made available in a forthcoming report.

### Patient involvement

Experiences of PEH and their views were sought from the outset of the development of the PHOENIx service and the trial^21^^,^^22^ with a qualitative investigation with both participants and stakeholders to assess the acceptability of the service. The PHOENIx pilot RCT trial intervention was devised in partnership with those with lived experience.

### Burden of treatment

Through consultation with its developer, the authors of the current study selected domain scales and slightly modified the original PETS measure (version 2.0), tailoring it for use in this population. PEH were not involved in this process. The original PETS measure was developed to assess treatment burden in patients with chronic health conditions requiring self-management.^19^ It has been extensively validated in patients with multimorbidity, including those with low income.^19^^,^^20^^,^^23^

Version 2.0 of the PETS consists of 60 items divided into 14 content domains (12 multi-item burden scales and two single-item indicators of medication bother): medicines (*n* = 7), medication side effect bother (*n* = 1), medication reliance bother (*n* = 1), medical information (*n* = 7), medical appointments (*n* = 6), monitoring health (*n* = 2), diet (*n* = 3), exercise and physical therapy (*n* = 4), interpersonal challenges (*n* = 4), medical and healthcare expenses (*n* = 5), difficulty with health services (*n* = 7), medical equipment (*n* = 2), role and social activity limitations due to self-management (*n* = 6), and physical and mental exhaustion due to self-management (*n* = 5). The modified version used in this study consisted of 52 items divided into 12 content domains. Differences between this version and the original are highlighted below.

The five items of the ‘medical and healthcare expenses’ domain were removed as the content is not relevant to a Scottish population where health care is free at point of access. The three items of the ‘diet’ domain were also removed, as this content was covered elsewhere in baseline data collection. As no single global treatment burden score is generated for the PETS, it is permissible for users to select for use only those domain scales that are most relevant to their study. One item from the ‘medical appointments’ domain was removed as it was deemed similar to a previous question (‘how easy/difficult is it to schedule medical appointments?’); this avoided repetition for the participants in an otherwise lengthy baseline assessment. Participants were also asked to categorise why it was difficult to attend medical appointments to gain information to identify barriers as to why participants may struggle to access their appointments. A single item was added to the ‘medical equipment’ domain to assess how easy/difficult it was to obtain medical equipment. This was added because PEH experience more barriers in accessing health care and this was seen as useful information to add when looking at treatment burden.

Scoring for the domains that were modified (that is, medical appointments and medical equipment) was adjusted slightly to account for the item changes, but followed the same general scoring procedure of the original PETS measure.^19^ Participants provided responses on either a four- or five-point categorical-ordered response scale, depending on the domain (for example, very easy to very difficult, or strongly agree to strongly disagree). As treatment burden can vary in terms of personal relevance, a ‘not applicable’ response option was available for some domains, and was coded as missing for the purposes of domain scoring. Raw scale scores were computed per guidelines defined by Eton *et al*^19^ then transformed to a standardised 0–100 scale with a higher score indicating higher treatment burden.

Aggregate summary scores for ‘workload and impact’ were also calculated.^23^ A workload summary score was calculated from the mean of the medical information, medications, medical appointments, and monitoring health domains; these are scales that indicate the difficulty of performing the work of self-management. An impact summary score was calculated from the mean of the role and social activity limitations and the physical and mental exhaustion domains as they are scales that indicate the impact of the workload of self-management on a person’s functioning and wellbeing.

### Missing data

Missing data for all domain scales were checked. PETS scoring does make allowances for some missing item data (that is, non-response or a response of ‘not applicable’ to an item). A domain scale score can still be calculated if >50% of the items within the domain are answered by the participant. This is made possible through prorated scoring that takes the mean of the non-missing items and assigns a raw score followed by standardisation to the 0–100 scale metric. The domain-level missing data are reported in the Results section (Table 1).

**Table 1.** Descriptive statistics of mean standardised PETS scores^a^

**Domain**	**Mean score (95% CI)**	**SD**	**Missing domain data, *n*/*N* (%)**
**Medical information**	43.4 (39.5 to 47.2)	4.2	9/123 (7.3)
**Medications**	29.1 (24.3 to 34.0)	5.2	8/123 (6.5)
**Medical appointments**	56.0 (39.2 to 56.5)	8.4	1/123 (0.8)
**Monitoring health**	53.8 (46.6 to 60.9)	0.8	3/123 (2.4)
**Role and social activity limitations due to self-management**	64.0 (60.3 to 67.6)	3.5	0
**Physical and mental exhaustion due to self-management**	79.5 (75.5 to 83.6)	3.3	0
**Exercise and physical therapy**	61.4 (36.2 to 86.5)	15.8	0
**Difficulties with medical equipment**	22.3 (21.5 to 23.0)	0.3	32/123 (26.0)
**Interpersonal challenges**	53.2 (28.7 to 77.7)	15.4	0
**Difficulty with healthcare services**	57.9 (42.3 to 73.6)	16.9	8/123 (6.5)
**Medication reliance bother**	47.6 (39.7 to 55.6)	43.0	6/123 (4.9)
**Medication side effect bother**	23.0 (16.7 to 29.4)	34.4	6/123 (4.9)
**Workload summary score**	45.8	12.5	—
**Impact summary score**	71.8	11.0	—

a *Scores are 0–100 with 100 being the highest treatment burden. Missing domain data are number of participants who answered >50% of the items as ‘n/a’ or did not provide an answer. PETS = Patient Experience with Treatment and Self-management. SD = standard deviation.*

## RESULTS

### Baseline characteristics

Demographic and clinical characteristics of participants are shown in Table 2. Mean age of participants was 42.1 years, 71.5% were male, and 99.2% were of White ethnicity. The majority (91.1%) had >5 chronic conditions. The average number of long-term conditions (LTCs) per participant (including physical and mental health conditions) was 8.5, with an average number of three problem drugs used per participant and 91.9% of participants reporting at least one mental health condition.

**Table 2.** Baseline characteristics of the 123 participants who responded to PETS recruited into PHOENIx after overdose study 2021

**Characteristic**	**PHOENIx (*n* = 123), *n* (%)**
**Age, years, mean (SD)**	42.1 (8.4)

**Sex, male**	88 (71.5)

**Ethnicity, White**	122 (99.2)

**GP registered**	104 (84.6)

**‘Open to’ alcohol and drug recovery service**	112 (91.1)

**‘Open to’ mental health team**	42 (34.1)

**Number of long-term health conditions**	
0–1	0 (0)
2–4	11 (8.9)
5–8	56 (45.5)
9–16	56 (45.5)

**Any mental health condition (self-reporting and information from clinical records)**	113 (91.9)

**Average number of LTCs per participant**	8.5

**Most commonly used illicit drugs**	
Street valium^a^	107 (87.0)
Heroin	74 (60.2)
Cocaine	73 (59.3)

**Average number of illicit drugs used per participant**	3.0

a *Derivative of benzodiazepine. LTC = long-term condition. PETS = Patient Experience with Treatment and Self-management. PHOENIx = Pharmacist Homeless Outreach Engagement Non-medical Independent prescribing Rx. SD = standard deviation.*

### Burden of treatment

Table 1 shows the mean standardised scores for each PETS domain. Participants scored highest on the physical and mental exhaustion due to self-management (mean 79.5) and the role and social activity limitations due to self-management (mean 64.0) domains, demonstrating a striking impact of self-management on functioning and wellbeing. Mean scores were also high for exercise and physical therapy (61.4) and difficulty with healthcare services (57.9). Mean scores were lowest for the difficulties with medical equipment domain (22.3).

The mean impact summary score (71.8) was higher than the mean workload summary score (45.8), perhaps indicating that the accumulated impact of the workload of self-care may be more meaningful to people than how difficult it is to perform. As shown in the last column of Table 1, the domain scale with the highest level of missing data was ‘difficulties with medical equipment’ with 26.0% missing. All other scales were below 10% missing.

Participants were asked to characterise why they found it difficult to attend appointments (Table 3). In total, 73 participants (59.3%) reported some difficulty attending appointments, the most common reason cited being a lack of money (58.9%, *n* = 43/73).

**Table 3.** Number of patients who found it difficult to attend appointments and the reasons given^^a^,^b^^

**Characteristic**	***n* (%)**
**Participants who found it difficult to attend appointments**	73 (59.3)

**Reasons given**	
Money	43 (58.9)
Help/support	2 (2.7)
Eyesight/health care	2 (2.7)
Motivation/mood	3 (4.1)
No transport	6 (8.2)
No reason given	17 (23.3)

a *This was a free-text option.*

b *In total, 73 of 123 responded to these questions.*

## DISCUSSION

### Summary

This study shows that PEH have high levels of multimorbidity with each participant having an average of 8.5 LTCs, high levels of problem drug use, as well as complex health needs, and high treatment burden that affects physical, mental, and social wellbeing. They had extremely high scores on the PETS impact domains of physical and mental exhaustion because of self-management and role and social activity limitations, highlighting the major adverse effects of the work of self-management on functioning and wellbeing.

### Strengths and limitations

This is the first study, to the authors’ knowledge, to focus on treatment burden in PEH, adding knowledge about a marginalised patient group. It used a previously validated measure of treatment burden, the PETS, which was interviewer-administered in person to ensure understanding and reduce the number of missing responses. However, the study participants were restricted to one country (Scotland) and most participants were White males, so findings are not necessarily generalisable across different marginalised populations. The current study did not include input from PEH when tailoring the PETS for administration in this population, which is a limitation. Although the amount of missing domain scores was not significant, any missing data have the potential to skew scores.

### Comparison with existing literature

Compared with other recent studies that have used the PETS (Table 4) where the focus was patients with multimorbidity, chronic heart failure, or hypertension, the sample in the present study showed markedly higher scores in all burden domains. Most striking are differences in the mean physical and mental exhaustion scores between the present study (79.5) and the prior studies (28.7, 34.12, and 22.8, respectively).^23^^–^^25^ Participants of this study were quite a bit younger (mean age 42.1 years) than those in the other studies (mean ages >60 years).^23^^–^^25^ Although these differences in treatment burden are noteworthy, caution must be exercised when comparing scores given study differences in methods as well as demographics, medical characteristics, and social circumstances of the samples. In this study, two domain scales of the PETS were slightly modified — medical appointments and difficulties with medical equipment — although in each case they differ by only a single item from the original measure. However, it is still useful to compare results, and the current results suggest that having multimorbidity in the context of homelessness, particularly when also having problem drug use, may have a profound impact on a person’s wellbeing and ability to effectively self-manage.

**Table 4.** Comparison of PHOENIx results with three other trials using PETS to measure treatment

**Characteristic**	**Current study, PHOENIx at baseline**	**Eton *et al* (2020), PETS at baseline^23^**	**Nordfonn *et al* (2021)^24^**	**Rogers *et al* (2021)^25^**
**Participants, *n***	123	365	125	254

**Focus of article**	Homeless health	Patients with multiple LTCs	Chronic heart failure	Hypertension

**Age of participants, years, mean**	42.1	62.1	67	67

**PETS score, mean (SD)**				
Medical information	43.4 (4.2)	25.6 (19.8)	34.65 (18.24)	24.7 (19.2)
Medications	29.1 (5.2)	15.9 (16.9)	16.16 (17.36)	9.1 (16.3)
Medical appointments	56.0 (8.4)	21.6 (21.7)	16.87 (16.85)	10.4 (20.0)
Monitoring health	53.8 (0.8)	32.3 (26.2)	30.86 (21.13)	16.1 (23.1)
Exercise or physical therapy	61.4 (15.8)	48.8 (24.6)	—	—
Medical equipment	22.3 (0.3)	—	—	—
Interpersonal challenges	53.2 (15.4)	—	14.23 (18.34)	—
Difficulty with healthcare services	57.9 (16.9)	30.3 (20.2)	34.57 (19.01)	30.0 (19.8)
Role and social activity limitations	64.0 (3.5)	20.0 (25.7)	27.53 (24.87)	15.0 (23.0)
Physical and mental exhaustion	79.5 (3.3)	28.7 (25.7)	34.12 (21.00)	22.8 (24.4)
Medication side effect bother	23.0	18.2 (25.7)	—	15.9 (25.3)
Medication reliance bother	47.6	—	—	20.5 (30.5)

*LTC = long-term condition. PETS = Patient Experience with Treatment and Self-management. PHOENIx = Pharmacist Homeless Outreach Engagement Non-medical Independent prescribing Rx. SD = standard deviation.*

These findings extend understanding about the complexity and impact of multimorbidity on treatment and self-management for PEH.^7^ Previous studies have reported that PEH understand what it is to be healthy, but feel they have little control over this^26^ and meet multiple barriers within the healthcare system, including feeling stigmatised by both staff and the healthcare system, which can be inflexible and is not tailored to their health and social needs.^26^^,^^27^ Studies have also shown that experiencing homelessness, or having poor living conditions, has a negative impact on quality of life and a person’s ability to improve their health and access services.^26^^,^^28^ High scores for mental and physical exhaustion may reflect the unique workload experienced by PEH who have difficulty accessing services while being itinerant in often challenging life circumstances.^29^ This new evidence on the treatment burden of PEH highlights the difficulty for participants trying to access services. Research has shown that PEH report healthcare professionals lack understanding of the complexities of their condition, which prevents them from accessing appropriate health care.^30^ A further study looking at how to engage people who use drugs in health care has shown that it takes time to build trust between healthcare workers and patients, and that continuity of care is key.^31^

There is a gap in the literature with regards to treatment burden among marginalised patient groups,^11^ and this study provides novel data to address this gap. It adds to understanding about why PEH struggle to engage with primary care services. Literature on people with chronic illness has acknowledged that accumulated treatment burden can lead to patients being overwhelmed and having poor health outcomes.^32^

To the authors’ knowledge, there are no other studies of treatment burden in the PEH population; it is not possible to directly compare the results with similar populations.

### Implications for research and practice

An assessment of treatment burden is an important person-centred outcome to help compare the effectiveness of interventions in PEH, and merits inclusion in future trials as an outcome measure. Understanding more about the treatment burden of PEH can help shape how to better tailor healthcare interventions to this socially marginalised, multimorbid group. Other than PHOENIx (which is under evaluation through the pilot RCT described above), there are currently no specific interventions in the UK, to the authors’ knowledge, to reduce the treatment burden workload of PEH. Tailored, intersecting outreach services, such as the PHOENIx intervention, may bridge a gap, while PEH are navigating poor health and high treatment burden where mainstream services may not meet their needs.

In conclusion, this study provides, to the authors’ knowledge, the first ever data on treatment burden in PEH. Results indicate that there is a profound negative impact on the overall functioning and wellbeing of this population because of their need to self-manage multimorbidity. This adds to barriers in patients accessing or participating in their health and self-care, and this study provides insights about why tailored input is needed, and what aspects can be targeted through multifaceted support interventions.

## Data

All data used during the study are available on reasonable request from the corresponding author. The Patient Experience with Treatment and Self-management (PETS), including all modified, translated, and adapted versions of it, are protected by copyright, © 2016, 2020 Mayo Foundation for Medical Education and Research. All rights reserved. Permission to use the PETS can be sought from David T Eton (dteton99@gmail.com).

## References

[1] Scottish Government (2021). Homelessness in Scotland: 2020 to 2021. https://www.gov.scot/publications/homelessness-scotland-2020-2021.

[2] Bowen M, Marwick S, Marshall T (2019). Multimorbidity and emergency department visits by a homeless population: a database study in specialist general practice. Br J Gen Pract.

[3] Hewett N, Hiley A, Gray J (2011). Morbidity trends in the population of a specialised homeless primary care service. Br J Gen Pract.

[4] National Records of Scotland (2021). Homeless deaths 2019. https://www.nrscotland.gov.uk/news/2021/homeless-deaths-2019.

[5] National Records of Scotland (2022). Drug-related deaths in Scotland in 2021. https://www.nrscotland.gov.uk/files//statistics/drug-related-deaths/21/drug-related-deaths-21-report.pdf.

[6] McDonald SA, McAuley A, Hickman M (2021). Increasing drug-related mortality rates over the last decade in Scotland are not just due to an ageing cohort: a retrospective longitudinal cohort study. Int J Drug Policy.

[7] Bramley G, Fitzpatrick S, Wood J (2019). Hard edges Scotland. https://lankellychase.org.uk/wp-content/uploads/2019/06/Hard-Edges-Scotland-full-report-June-2019.pdf.

[8] Eton DT, Ramalho de Oliveira D, Egginton JS (2012). Building a measurement framework of burden of treatment in complex patients with chronic conditions: a qualitative study. Patient Relat Outcome Meas.

[9] Mair FS, May CR (2014). Thinking about the burden of treatment. BMJ.

[10] Gallacher K, May CR, Montori VM, Mair FS (2011). Understanding patients’ experiences of treatment burden in chronic heart failure using normalization process theory. Ann Fam Med.

[11] Mair FS, Gallacher KI (2017). Multimorbidity: what next?. Br J Gen Pract.

[12] Nathan D, Shippee NDS, May CR (2012). Cumulative complexity: a functional, patient-centered model of patient complexity can improve research and practice. J Clin Epidemiol.

[13] Sav A, King MA, Whitty JA (2015). Burden of treatment for chronic illness: a concept analysis and review of the literature. Health Expect.

[14] Barnett K, Mercer SW, Norbury M (2012). Epidemiology of multimorbidity and implications for health care, research, and medical education: a cross-sectional study. Lancet.

[15] Zeitler M, Williamson AE, Budd J (2020). Comparing the impact of primary care practice design in two inner city UK homelessness services. J Prim Care Community Health.

[16] Queen AB, Lowrie R, Richardson J, Williamson AE (2017). Multimorbidity, disadvantage, and patient engagement within a specialist homeless health service in the UK: an in-depth study of general practice data. BJGP Open.

[17] Lowrie R, Stock K, Lucey S (2021). Pharmacist led homeless outreach engagement and non-medical independent prescribing (Rx) (PHOENIx) intervention for people experiencing homelessness: a non-randomised feasibility study. Int J Equity Health.

[18] Lowrie F, Gibson L, Towle I, Lowrie R (2019). A descriptive study of a novel pharmacist led health outreach service for those experiencing homelessness. Int J Pharm Pract.

[19] Eton DT, Yost KJ, Lai J-S (2017). Development and validation of the Patient Experience with Treatment and Self-management (PETS): a patient-reported measure of treatment burden. Qual Life Res.

[20] Eton DT, Linzer M, Boehm DH (2020). Deriving and validating a brief measure of treatment burden to assess person-centered healthcare quality in primary care: a multi-method study. BMC Fam Pract.

[21] Johnsen S, Cuthill F, Blenkinsopp J (2021). Outreach-based clinical pharmacist prescribing input into the healthcare of people experiencing homelessness: a qualitative investigation. BMC Health Serv Res.

[22] Johnsen S, Cuthill F, Blenkinsopp J (2019). Qualitative evaluation of clinical pharmacist prescribing input into the care of people experiencing homelessness.

[23] Eton DT, Lee MK, St Sauver JL, Anderson RT (2020). Known-groups validity and responsiveness to change of the Patient Experience with Treatment and Self-management (PETS vs. 2.0): a patient-reported measure of treatment burden. Qual Life Res.

[24] Nordfonn OK, Morken IM, Bru LE (2021). Burden of treatment in patients with chronic heart failure — a cross-sectional study. Heart Lung.

[25] Rogers EA, Abi H, Linzer M, Eton DT (2021). Treatment burden in people with hypertension is correlated with patient experience with self-management. J Am Board Fam Med.

[26] Mc Conalogue D, Maunder N, Areington A (2019). Homeless people and health: a qualitative enquiry into their practices and perceptions. J Public Health (Oxf).

[27] Purkey E, MacKenzie M (2019). Experience of healthcare among the homeless and vulnerably housed a qualitative study: opportunities for equity-oriented health care. Int J Equity Health.

[28] Lewer D, Aldridge RW, Menezes D (2019). Health-related quality of life and prevalence of six chronic diseases in homeless and housed people: a cross-sectional study in London and Birmingham, England. BMJ Open.

[29] Lowrie R, McPherson A, Mair FS (2023). Baseline characteristics of people experiencing homelessness and drug overdose: PHOENIx pilot RCT. Harm Reduct J.

[30] Gunner E, Chandan SK, Marwick S (2019). Provision and accessibility of primary healthcare services for people who are homeless: a qualitative study of patient perspectives in the UK. Br J Gen Pract.

[31] Woolhouse S, Brown JB, Thind A (2011). ‘Meeting people where they’re at’: experiences of family physicians engaging women who use illicit drugs. Ann Fam Med.

[32] May CR, Eton DT, Boehmer K (2014). Rethinking the patient: using burden of treatment theory to understand the changing dynamics of illness. BMC Health Serv Res.

